# Supplementary material for: Mutations in PCYT2 disrupt etherlipid biosynthesis and cause a complex hereditary spastic paraplegia
Source: Brain. 2019 Oct 22;142(11):3382–97. doi: 10.1093/brain/awz291 (PMC6821184; doi:10.1093/brain/awz291)
Supplement: awz291_Supplementary_Data [file awz291_supplementary_data.zip › awz291-Suppl_data/Supplementary_Data4.zip!/Supplementary information 3 - fibroblast lipidomics.zip!/Fibroblast lipidomics/Controle vs Patient1/Statistical plots and data/TG species/Heatmap.pdf]

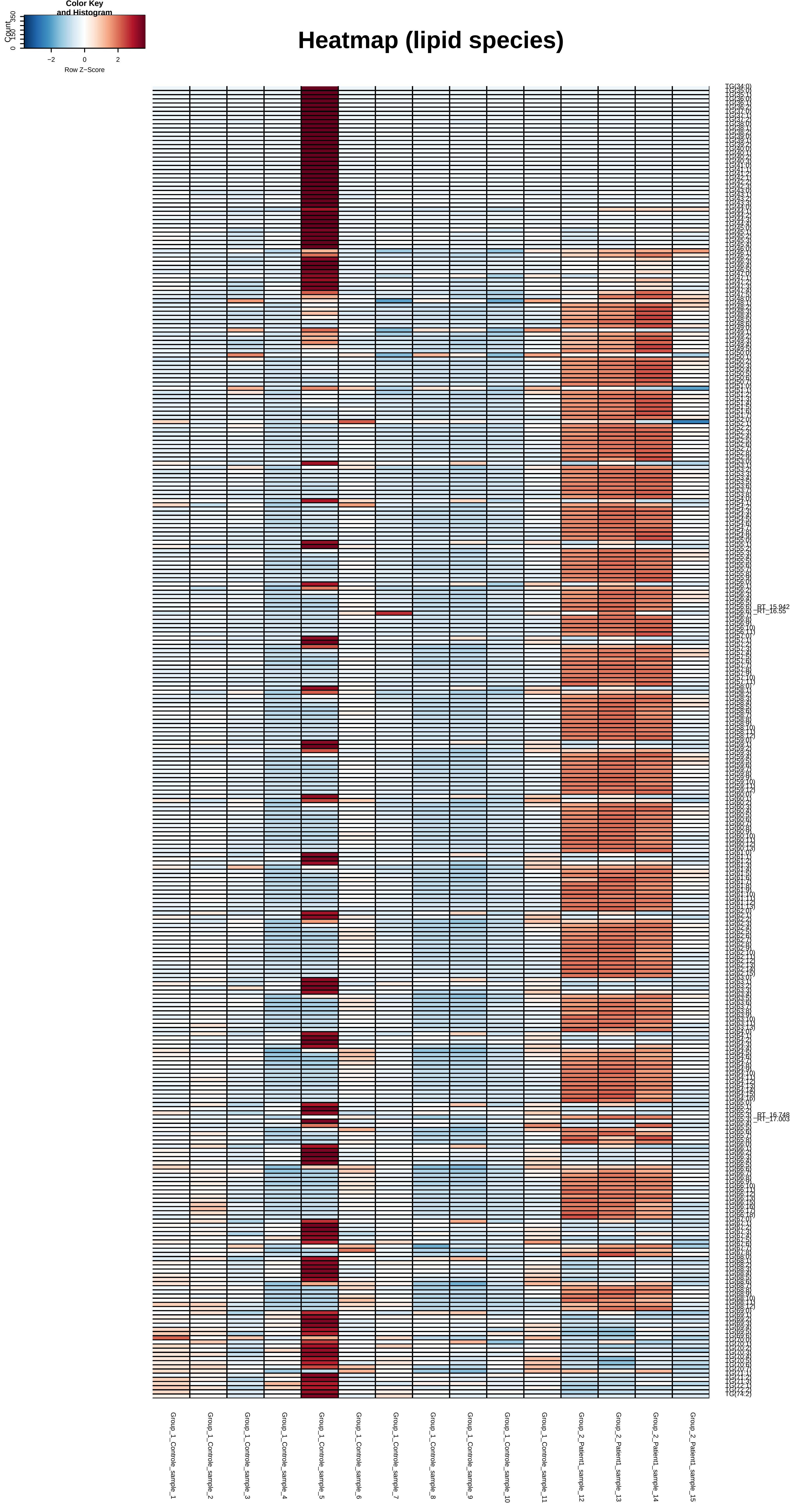
Color Key
and Histogram
Count
Row Z−Score
−2
0
2
0
150
350
Heatmap (lipid species)
TG(34:0)
TG(74:2)
RT_15.942
RT_16.55
RT_16.748
RT_17.003
Group_1_Controle_sample_1
Group_1_Controle_sample_2
Group_1_Controle_sample_3
Group_1_Controle_sample_4
Group_1_Controle_sample_5
Group_1_Controle_sample_6
Group_1_Controle_sample_7
Group_1_Controle_sample_8
Group_1_Controle_sample_9
Group_1_Controle_sample_10
Group_1_Controle_sample_11
Group_2_Patient1_sample_12
Group_2_Patient1_sample_13
Group_2_Patient1_sample_14
Group_2_Patient1_sample_15
